# Genetic Aspects of Bone Remodelling in Children under One Year of Age in the Kazakh Population

**DOI:** 10.34763/jmotherandchild.20252901.d-25-00005

**Published:** 2025-07-02

**Authors:** Akmaral Zhumalina, Irina Kim, Balash Tusupkaliev, Mairamkul Zharlykasinova, Svetlana Sakhanova

**Affiliations:** Department of Pediatric Diseases No. 1 with Neonatology, West Kazakhstan Marat Ospanov Medical University, Aktobe, Republic of Kazakhstan; Scientific-Practical Center, West Kazakhstan Marat Ospanov Medical University, Aktobe, Republic of Kazakhstan

**Keywords:** bone metabolism, vitamin D status, genotype, ethnic characteristics, bone tissue density

## Abstract

**Background:**

This study investigates genetic markers, such as polymorphisms in the vitamin D receptor (VDR) and receptor activator of nuclear factor-kappa B ligand (RANKL) genes, to determine if they can serve as prognostic indicators for the development of bone-tissue pathologies in childhood.

**Material and Methods:**

The study included 104 healthy children aged from birth to 12 months. Genetic testing was conducted to identify polymorphisms in the VDR and RANKL genes.

**Results:**

78% of the 104 children from the Kazakh population showed a decrease in the level of vitamin D, with particularly promising results in infants seven to 12 months old. Indicators of total calcium and phosphorus in children were uninformative for bone-metabolism analysis. The homozygous C/C type according to RANKL rs9594759 was detected in 17% of children; the homozygous T/T variant according to RANKL rs9594738 was detected in 28%; the homozygous T/T according to VDR rs2228570 was detected in 17%; and the homozygous A/A according to VDR rs2228570 was detected in 4%. These variant polymorphisms are associated with reduced bone density. RANKL rs9594738 and RANKL rs9594759 have shown a moderate connection with vitamin D serum concentration.

**Conclusion:**

A relatively strong relationship was found between the T/T and C/T genotypes of the VDR gene and the concentration of vitamin D falling below the norm, and there is a direct relationship between vitamin D levels and bone pathology risk in children.

## 1. Introduction

Recently, several researchers have focused on the genetic-molecular mechanisms of bone tissue remodelling and their control [1, 2]. In Western Kazakhstan, 70% of 396 healthy 12-to-17-year-olds were found to have osteopenia [3, 4]. In children, especially those under one year old, biological processes occurring in the background of linear growth and differentiation of bone tissue promote remodelling [5], and from the foetal stage to adulthood, bone tissue undergoes extensive metabolic remodelling [6, 7]. J.P. Brown et al. [8] found that calcium-phosphorus homeostasis and bone metabolism markers, including vitamin D, calcitonin, and osteocalcin, affect intraosseous processes. Osteoblasts synthesise osteocalcin (OC), a bone matrix protein that is one of the most reliable biochemical markers of osteosynthesis and bone tissue exchange [9,10,11].

Vitamin D receptors (VDR) and receptor activator of nuclear factor-kappa B ligand (RANKL) play an important role in the pathogenesis of osteoporosis, as there is a correlation between the allelic polymorphism of the candidate gene and bone remodelling markers [12,13,14]. T. Ono et al. describe RANKL as an osteoblast-synthesised protein that induces monocytic-macrophage cells to differentiate into osteoclasts [15]. Abnormalities in this ligand and gene polymorphism lead to osteopetrosis and osteoporosis, but RANKL also plays a role in organogenesis, immune response formation, and thermoregulation [16,17,18]. Pathological fractures, secondary hypoparathyroidism, and vitamin D-resistant rickets are linked to the single-nucleotide BsmI polymorphism (VDR rs1544410), which affects osteogenesis and vitamin D sensitivity. Although it affects several metabolic pathways in immune response and oncology, this transactive regulatory transcription factor receptor is specifically implicated in mineral metabolism [19,20,21,22].

Examining calcium-phosphorus metabolism indicators, calcium-regulating hormones, bone remodelling markers, and genetic markers in the blood serum of healthy children across various ages can help predict metabolic alterations in bone tissue. However, no research has previously been performed on Kazakh children under one year of age; this study aims to rectify this by investigating the relationship between bone remodelling processes and the genotype of the VDR and RANKL genes.

## 2. Materials and methods

This study was conducted by the Department of Pediatric Diseases No. 1 with Neonatology, West Kazakhstan Marat Ospanov Medical University. The study included 104 healthy children aged from birth to 12 months, inclusive, who had prior informed consent signed by parents or guardians in accordance with the current legislation of the Republic of Kazakhstan. Factors that excluded children from the study included hereditary musculoskeletal diseases; severe chronic somatic diseases; disability; treatment with vitamin D therapy; parental refusal; premature birth; and age over one year old.

Aktobe's biochemistry lab assessed bone tissue remodelling indicators. Under fasting conditions, 5 ml of blood serum was collected in the procedure room using a vacuum system according to standard biochemical research procedures. After blood collection, activator gel was added and centrifuged at 1800 G for 10 min at room temperature. Serum samples under 0.5 ml were frozen at −20 °C and delivered to a centralised lab without harm according to their temperature regime. For the quantitative determination of bone metabolism markers, electro chemiluminescent two-site immunoassays were used for OC (intact and in fragments), vitamin D, calcium, phosphorus, and calcitonin. The examination of genetic markers identified four polymorphisms: VDR rs1544410 and rs2228570, and RANKL rs9594738 and rs9594759. Whole blood in 2 ml ethylenediaminetetraacetic acid (EDTA) was used for the study, followed by the polymerase chain reaction (PCR) method “in real time” using adjacent fluorescently-labelled samples (kissing probes) and melting-curve analysis. Technical support came from the detection amplifier DTprime.

Statistical processing involved descriptive analysis of quantitative data in groups and group comparison on a personal computer using Microsoft Excel and EpiData database, as well as Statistica 10.0 or IBM SPSS Statistics 25.0 software. Arithmetic mean, median, mode, standard error, and standard deviation were calculated. Statistical significance was defined as *p* < 0.05. The statistical significance of relative indicators was determined using the Pearson consensus criterion (χ^2^), while Fisher's exact and Cramer's V tests determined the relationship between variables.

## 3. Results

At the beginning of the study, the vitamin D supply of the examined children was assessed; this is shown in Table 1. The children were redistributed into the following categories: those with normal vitamin D concentration, with 30 to 75 ng/ml in their blood serum; those with insufficient vitamin D, with 20 to 30 ng/ml; and those with vitamin D deficiency, with less than 20 ng/ml. Children were also redistributed according to their age; Group 1 consisted of new-borns (0–28 days of life), Group 2 consisted of children aged one to six months, and Group 3 comprised children aged 7–12 months.

**Table 1.** Vitamin D availability in the examined children

**25(OH)D ng/ml**	**New-borns (0-28 days)**	**1–6 months**	**7–12 months**	***p***
Norm	3 (6.7%)	4 (14.9%)	14 (43.75%)	0.001
Insufficiency	3 (6.7%)	7 (25.9%)	8 (25%)	
Deficit	39 (86.6%)	16 (59.2%)	10 (31.25%)	0.05

*Source:* created by the authors.

Group 1 showed vitamin D insufficiency, with only 6.7% of the children having normal levels. Blood vitamin D levels in 86.6% of the 45 children had dropped, indicating insufficiency. Vitamin D deficiency was found in 85.2% of the children in Group 2, with 59.2% meeting the criteria. 14.9% of children had normal vitamin levels.

In Group 3, these indicators were 56.25%, 31.25%, and 43.75%, which has a more positive age dynamic but does not allow for a definitive assessment, as vitamin D is important from birth and must be provided by breastfeeding and/or exogenous 25-hydroxycholecalciferol in the form of prescribed medications.

Vitamin D (25-OH-D) blood levels fluctuate throughout childhood. Infants in the first year of life are characterised by low median vitamin D concentrations and moderate interquartile variability. The preschool group (two to six years) shows increased mean and median vitamin D, but also displays more measurement variability. The lower and higher quartiles vary more, with median vitamin D concentrations peaking in the older age group (seven to 12 years). Note the seven-to-12-year-old group's unusually high values, which exceed the normal distribution's upper limit. The original data falls into a right-skewed distribution, with outliers at higher concentrations, because all age groups' arithmetic mean values exceed the medians. This age-related increase in vitamin D indicates a need for more research on the effects of diet, sun exposure, and vitamin-mineral supplementation. A comparison of vitamin D levels in three groups showed significant differences between Groups 1 and 3 (*p* = 0.001) as well as between 2 and 3 (*p* = 0.05) (Table 2).

**Table 2.** Indicators of total calcium and phosphorus depending on age

**Dependent: Total calcium=2.25–2.75 mmol/l**	**p-values (2-sided) for the set comparisons; total calcium=2.25–2.75 mmol/l (beginning); group (independent) variable: age; Kruskal-Wallis H (N=104)=0.1979832; p=0.9058**		
	**Group 1 (R: 32.614)**	**Group 2 (R: 32.462)**	**Group 3 (R: 30.167)**
Group 1		1	1
Group 2	1		1
Group 3	1	1	

**Dependent: Phosphorus=1.45–2.16 mmol/l**	**p-values (2-sided) for the set comparisons; phosphorus=1.45–2.16 mmol/l (beginning); group (independent) variable: age; Kruskal-Wallis H (N=104)=3.287534; p=0.1933**		
	**Group 1 (R: 35.743)**	**Group 2 (R: 27.462)**	**Group 3 (R: 27.2)**
Group 1		0.492712	0.392994
Group 2	0.492712		1
Group 3	0.392994	1	

*Source:* created by the authors.

Table 2 shows that all the children's total calcium levels were within the age norm (2.8 ± 0.104 mmol/l) and did not differ from reference values. The average blood phosphorus concentration in the children was 1.4 ± 0.2 mmol/l, meeting reference norms. Analysis by age revealed significantly higher serum phosphorus concentration in infants aged 0 to 6 months (2.52 ± 0.2 mmol/l, *p* < 0.05) compared to those aged 7 to 12 months. Despite this age-related difference in phosphorus levels, no substantial alterations in calcium-phosphorus homeostasis were observed, indicating overall stability in mineral metabolism during infancy. The research shows that in children under one year old, phosphorus and calcium levels in blood serum do not correspond to vitamin D levels or bone metabolism; this may imply asymptomatic vitamin D deficiency. The study revealed an average calcitonin level of 4.369 ± 0.168 ng/ml in the children (Table 3).

**Table 3.** Calcitonin indicators depending on age

**Dependent: Calcitonin=0–9.5 pg/ml**	**p-values (2-sided) for the set comparisons; calcitonin=0–9.5 pg/ml (beginning); group (independent) variable: age; Kruskal-Wallis H (N=104)=4.171448; p=0.1242**		
	**Group 1 (R: 31.7)**	**Group 2 (R: 39.846)**	**Group 3 (R: 25.9)**
Group 1		0.513694	0.915658
Group 2	0.513694		0.133990
Group 3	0.915658	0.133990	

*Source:* created by the authors.

The average serum parathyroid hormone level was 37.05 ± 1.425 ng/ml. Except for those in Group 3 with biochemical hyperparathyroidism, parathyroid hormone and calcitonin parameters were within reference levels by age. Group 2 had greater parathyroid hormone levels than Group 3 (77 ± 1.425 ng/ml vs. 36.1 ± 1.086 ng/ml, *p* < 0.001). Although neither calcitonin nor parathyroid hormone was connected to vitamin D or bone metabolism in infants under 1 year old, these metabolic indicators were beneficial. Children in group 1 had lower OC (*p* = 0.05) than groups 2 and 3, indicating vitamin D deficiency and suggesting that OC may be a better index of bone turnover (Table 4).

**Table 4.** Indicators of parathyroid and osteocalcin hormones depending on age

**Dependent: Parathyroid hormone=15–65 pg/ml**	**p-values (2-sided) for the set comparisons; parathyroid hormone=15–65 pg/ml (beginning); group (independent) variable: age; Kruskal-Wallis H (N=104)=1.05037; p=0.5914**		
	**Group 1 (R: 30.986)**	**Group 2 (R: 29.923)**	**Group 3 (R: 36.167)**
Group 1		1	1
Group 2	1		1
Group 3	1	1	

**Dependent: Osteocalcin=2.8–41 ng/ml**	**p-values (2-sided) for the set comparisons; osteocalcin=2.8–41 ng/ml (beginning); group (independent) variable: age; Kruskal-Wallis H (N=104)=3.25855; p=0.1961**		
	**Group 1 (R: 28.671)**	**Group 2 (R: 36.154)**	**Group 3 (R: 36.167)**
Group 1		0.026506	0.05553
Group 2	0.026506		1
Group 3	0.05553	1	

*Source:* created by the authors.

Next was a molecular genetic analysis of the 104 children to determine VDR rs1544410, VDR rs2228570, RANKL rs9594738, and RANKL rs9594759 gene polymorphisms. The BsmI polymorphism (A < G) in the VDR gene rs1544410 is autosomal dominant, affecting both men and women equally. In over 50% of children, a single mutant variant from one parent could cause the disease to manifest. Variant-A carriers of this polymorphism are at risk of developing low bone mineral density, which impedes bones' mechanical characteristics and increases pathological fracture risk.

VDR rs 1544410 polymorphism carriers are more likely to have such problems. Low bone mineral density is reduced by vitamin G. The VDR gene rs 1544410 was found in 17% of children. Variant A/A is found in 17%, G/A in 31%, and G/G in 52%.

VDR rs2228570 (C<T) is derived from a single-nucleotide substitution of ‘C‘ for ‘T‘ on chromosome 12. Carriers of the T variant have an increased risk of developing low bone mineral density, while the C variant reduces the risk of low bone mineral density. VDR rs2228570 had a mutated variant in 4% of subjects. The distribution of allelic variants of VDR rs2228570 polymorphism among the examined children shows that variant C/C occurs in 76% of children, C/T in 20%, and T/T in 4%.

Carriers of the variant-T polymorphism of the RANKL rs9594738 gene (C < T) have an increased susceptibility to changes in bone mineral density, while carriers of the variant-C polymorphism are not associated with such a risk. Mutated RANKL rs9594738 was found in 28% of the subjects. The distribution of allelic variants of the RANKL rs9594738 polymorphism among the children in the study demonstrates that the C/C variant occurs in 57% of children, C/T occurs in 15%, and T/T is observed in 28%.

Carriers of the variant-T polymorphism of the RANKL gene rs9594759 (C > T) have an increased susceptibility to changes in bone mineral density, while carriers of the variant C polymorphism are not associated with this risk. The mutated RANKL variant rs9594759 was detected in 28% of the children examined in this study. The distribution of allelic variants of the RANKL rs9594759 polymorphism among the examined children demonstrates that the C/C variant occurs in 57% of children, the C/T in 15%, and the T/T in 28%.

Vitamin D levels showed considerable variation across different genotypes. For the RANKL rs9594759 polymorphism, vitamin D concentrations in the first group were 20% in children with the C/C genotype, 18.2% in those with the C/T genotype, and 29.4% in those with the T/T genotype. In the second group, the levels were 22% for C/C, 3% for C/T, and 29.4% for T/T. In the third group, the concentrations were 58% for C/C, 78.8% for C/T, and 41.2% for T/T.

Regarding the RANKL rs9594738 polymorphism, the first group exhibited vitamin D levels of 22.8% for C/C, 25% for C/T, and 6.6% for T/T. In the second group, the values were 19.2% for C/C, 3% for C/T, and 0% for T/T. The third group showed levels of 57.8% for C/C, 43.3% for C/T, and 93.3% for T/T.

The VDR rs2228570 genotype also had a significant impact on vitamin D levels. In the first group, vitamin D levels were 13.4% for C/C, 29% for C/T, and 29.4% for T/T. In the second group, the values were 21.7% for C/C, 3% for C/T, and 23.5% for T/T. In the third group, vitamin D concentrations were 65.3% for C/C, 64.5% for C/T, and 47% for T/T.

The children with the VDR rs1544410 genotype demonstrated considerable variability in their vitamin D levels. In the first group, children with the G/G genotype had vitamin D levels of 40%; those with the G/A genotype had 17%; and those with the A/A genotype had 0%. In the second group, the levels were 15% for G/G, 3% for G/A, and 0% for A/A. In the third group, the vitamin D levels were 45% for G/G, 64% for G/A, and 100% for A/A. These findings highlight the significant influence of genetic variation on children's vitamin D levels. Understanding these genetic factors can aid in developing personalized strategies to manage vitamin D deficiency more effectively.

The correlation between genotype and vitamin D concentration in the children was calculated using Fisher's exact and Cramer's V tests. The null hypothesis was that vitamin D concentration did not impact bone system pathology risk, and the significance level was 0.05. Since Fisher's test (F) yielded a figure below the critical level of significance (F > 0.05 units), the relationship between the T/T and C/T genotypes of the VDR gene and the change in vitamin D concentration below the normal value can be considered strongly correlated with bone-system pathology in the children.

Fischer's criterion and Cramer's V indicate varying relationships between vitamin D levels and childhood genetic polymorphisms. For the RANKL rs9594738 polymorphism, the C/C and C/T genotypes were moderately associated with vitamin D levels, with a Cramer's V of 0.275 and a significant p-value of 0.043. This association is average. C/C and T/T genotypes were weakly linked (Cramer's V = 0.147, p = 0.481). Vitamin D concentrations were found to be strongly related in comparison with a Cramer's V of 0.437 and a p-value of 0.008.

For the RANKL rs9594759 polymorphism, vitamin D levels are moderately correlated with genotypes C/C and C/T, with a Cramer's V of 0.306 and a significant p-value of 0.033. C/C and T/T comparisons, however, were inconsequential (Cramer's V = 0.041, p = 0.93). A Cramer's V of 0.411 and a p-value of 0.026 showed a stronger link between the C/T and T/T genotypes.

After conducting an analysis of the VDR rs2228570 polymorphism, it was discovered that the C/C and C/T genotypes had an average correlation with vitamin D levels (Cramer's V = 0.248, p = 0.047). The association between the C/C and T/T genotypes was weaker (Cramer's V = 0.194, p = 0.27), while the comparison between C/T and T/T showed an average connection (Cramer's V = 0.255, p = 0.208), although it was not statistically significant.

Lastly, for the VDR rs1544410 polymorphism, no significant relationship was found between vitamin D concentrations and the G/G, G/A, or A/A genotypes. The connection between G/G and G/A was categorized as average (Cramer's V = 0.225, p = 0.086), but it did not reach statistical significance. The relationships were weak between both G/G and A/A (Cramer's V = 0.163, p = 0.342) and G/A and A/A (Cramer's V = 0.411, p = 0.131), with no substantial evidence of a connection.

The study illustrates varying degrees of association between vitamin D levels and specific genetic polymorphisms, with some genotypes showing stronger connections than others. The findings suggest that genetic factors play a role in determining vitamin D status, but the nature of the relationship varies depending on the specific polymorphism under consideration.

## 4. Discussion

Compared to cases where there is vitamin D supplementation, vitamin D deficiency is associated with lower bone-mineral density, greater bone resorption activity, and higher fracture rates [23,24,25]. M.L. Tan et al. examined 19 studies on vitamin D supply in nursing mothers and infants, focussing on bone health [26]. A daily dose of 400 to 4,000 IU of vitamin D raised serum levels of 25-hydroxycholecalciferol and reduced both biochemical and radiological signs of rickets in the first six months of life. The evidence was unreliable, however; though the authors warn against hypercalcemia from hypervitaminosis, infants from birth to six years of age accept higher vitamin D doses without serious adverse reactions, and the prescription of preventative vitamin D thus should not be limited [27, 28].

S. Hurmuzlu Kozler et al. found that 83% of children were prescribed vitamin D supplements in the first year of life, and 28% between the ages of one and two years. This rate was higher, however, among families who had family doctors. This points to the importance of regular examinations for healthy children and of educating medical professionals about the proper duration and dosage of such therapies [29].

Exogenous vitamin D was found to be ineffective on skeletal maturity and bone health, however, in socioeconomically disadvantaged children who had other nutritional insufficiencies; this only drives home the importance of normalising a child's nutritional status [30,31,32,33,34,35]. Our study analysed the bone system using x-ray and anthropometric methods – procedures that could be employed in future studies. Additionally, 78% of children not taking vitamin D supplements showed some degree of vitamin D deficiency, regardless of their genetics. We thus advise normalising nutrition and 25-hydroxycalciferol intake for all children.

C. Medina-Gomez et al. found that RANKL expression in bones increases markedly from youth to adulthood depending on external environmental factors, and is associated with bone-mass loss, which can lead to bone diseases in old age [36]. Other studies have investigated RANKL and glycoprotein osteoprotegerin expression in lymphoblastic leukaemia along with vitamin D and K supplement use in children [37]; juvenile idiopathic arthritis [38]; postmenopausal osteoporosis in older women [39]; and secondary hyperparathyroidism [40].

J. Arid et al. found that 35% of 56 children with delayed teething had at least one permanent tooth [41]. PCR testing showed that the T allele in RANKL rs9594738 increases the likelihood of delayed teething in children and reduces their RANKL gene expression and the RANKL/OPG ratio, thus confirming the advantages of lower bone metabolism. O.V. Garmash also found genetic patterns in tooth state: polymorphisms of BsmI (VDR rs1544410) and RANKL gene rs9594738 (C > T) increase the risk of moderate and high-intensity caries, as do other genes (cytochrome P450, oestrogen receptor 1, and interleukin 1, 6, 10) [42]. These articles show connections between RANKL gene polymorphism and bone remodelling and other childhood and adult disorders, indicate a need for extensive follow-ups with children that have the genetic variations found in this study.

E. Jakubowska-Pietkiewicz et al. examined 395 children aged six to 18 years for VDR gene polymorphisms, including BsmI (rs1544410), FokI (rs2228570), ApaI (rs7975232), and Taq I (rs731236). They found that the ApaI and FokI alleles in children increase bone tissue density, bone mineral content, and ultrasound densitometry speed [43]. E.V. Bolshova et al. found that the VDR BsmI polymorphism had a 62.5% allele frequency for the G allele and 37.5% for the A allele, and that carrying the G allele increased the risk of growth hormone deficiency despite maintenance of normal calcium, phosphorus, and vitamin D levels [44]. Other study teams found similar results for FokI [45] and BsmI [46] polymorphisms using lumbar spine radiography. In a study of 100 healthy girls, R. Chowdhary et al. found that the homozygous polymorphic VDR allele was associated with severe secondary hyperparathyroidism and hypovitaminosis D, with a prevalence of 84.9% to 100% — similar to the prevalence of 70.3% to 100% found in this study [47].

Thus, a molecular genetic study of VDR (rs1544410, rs2228570) and RANKL (rs9594738, rs9594759) gene polymorphisms can classify children and adults according to their risk of decreased bone density and predict the development of other pathologies. This allows for the development of a preventive program to normalise serum vitamin D, correct calcium-phosphorus metabolism, and eliminate hyperparathyroidism with complications in the form of osteoporosis, pathological fractures and disability of the population.

## 5. Conclusions

The study revealed a high prevalence of vitamin D deficiency among Kazakh children under one year of age, highlighting the need for early interventions to prevent musculoskeletal abnormalities. Genetic polymorphisms of VDR and RANKL showed a significant correlation with vitamin D levels, indicating their role in the regulation of bone metabolism. Despite normal calcium and phosphorus levels, osteocalcin and parathormone were found to be correlated with vitamin status; this may serve as a useful marker for monitoring bone metabolism. The results highlight the importance of a personalised approach – taking into account genetic predisposition – in the prevention and treatment of vitamin D deficiency and the improvement of bone health in children.

The study's focus on Kazakh youngsters limits its applicability to other ethnic groups, and its sample size of 104 children may not completely capture population heterogeneity. The cross-sectional design makes causal linkages difficult to establish, and it ignores many environmental factors like nutrition and solar exposure. To improve reliability, future studies should cover other ethnic populations and use greater sample sizes. Advanced imaging and longitudinal vitamin D and bone health investigations will provide more information. Preventive efforts can be further enhanced with extensive environmental assessments, as well as interventional studies to test supplements and dietary changes. Hereditary counselling programmes should inform families about hereditary concerns to encourage early action for better bone health in their children.
